# Advanced hybrid-stabilized cinacalcet nanocrystals for enhanced oral bioavailability: formulation and integrated *in vitro/in vivo* evaluations

**DOI:** 10.3389/fphar.2026.1904508

**Published:** 2026-09-10

**Authors:** Rawan Gamal Eldin, Salma M. Mosleh, Osama Saher, Sadek Ahmed, Ahmed M. Fatouh

**Affiliations:** 1 Department of Pharmaceutics and Industrial Pharmacy, School of Pharmacy, Newgiza University, Giza, Egypt; 2 Department of Clinical Pharmacy, School of Pharmacy, Newgiza University, Giza, Egypt; 3 Department of Laboratory Medicine, Karolinska Institute, Stockholm, Sweden; 4 Department of Cellular Therapy and Allogeneic Stem Cell Transplantation (CAST), Karolinska University Hospital Huddinge and Karolinska Comprehensive Cancer Center, Stockholm, Sweden; 5 Department of Pharmaceutics and Industrial Pharmacy, Faculty of Pharmacy, Cairo University, Cairo, Egypt

**Keywords:** cinacalcet, high-speed homogenization, hybrid-stabilized nanocrystals, mixed factorial, pharmacokinetics, saturated solubility

## Abstract

**Introduction:**

Cinacalcet (CINA), a Biopharmaceutics Classification System (BCS) Class IV drug, exhibits limited oral bioavailability due to poor aqueous solubility and low intestinal permeability. We aimed to mechanistically enhance the oral bioavailability of CINA through the development of advanced hybrid-stabilized nanocrystals (HNCs) prepared through high-speed homogenization.

**Methods:**

A dual-stabilization strategy was implemented, combining a steric stabilizer (polyvinylpyrrolidone K90 or Pluronic® F-127) with a dispersing stabilizer (Tween® 80) to synergistically control nanocrystal formation, surface properties, and colloidal stability. A 2^1^ ×·3^2^ mixed factorial design was developed to investigate the effects of steric stabilizer type (Factor-A), total stabilizer concentration (Factor-B), and dispersing stabilizer contribution (Factor-C) on particle size (PS), poly-dispersity index (PDI), and saturated solubility (SS).

**Results:**

Numerical optimization identified an optimal formulation exhibiting a PS of 193.44 nm, a PDI of 0.34, and an SS of 2.04 mg/mL, corresponding to a 1.8-fold increase compared with unprocessed CINA (1.10 ± 0.02 mg/mL). Furthermore, the optimized formula exhibited a zeta potential (ZP) of −24.25 ± 0.64 mV, indicating good colloidal stability. Transmission electron microscopy confirmed the formation of discrete, uniformly distributed nanocrystals with well-defined nanoscale morphology. The optimized formulation demonstrated markedly enhanced dissolution behavior in simulated gastric (pH 1.2) and intestinal (pH 6.8) media, alongside satisfactory zeta potential and physical stability. *In vivo* pharmacokinetic evaluation following oral administration revealed a substantial enhancement in systemic exposure, with 10.36- and 7.76-fold increases in peak plasma concentration (Cmax) and area under the plasma concentration–time curve (AUC), respectively, compared to a conventional CINA suspension.

**Conclusion:**

Collectively, these findings highlight hybrid-stabilized nanocrystals as a promising strategy for improving the oral bioavailability of poorly soluble and poorly permeable drugs.

## Graphical Abstract

**Figure:**
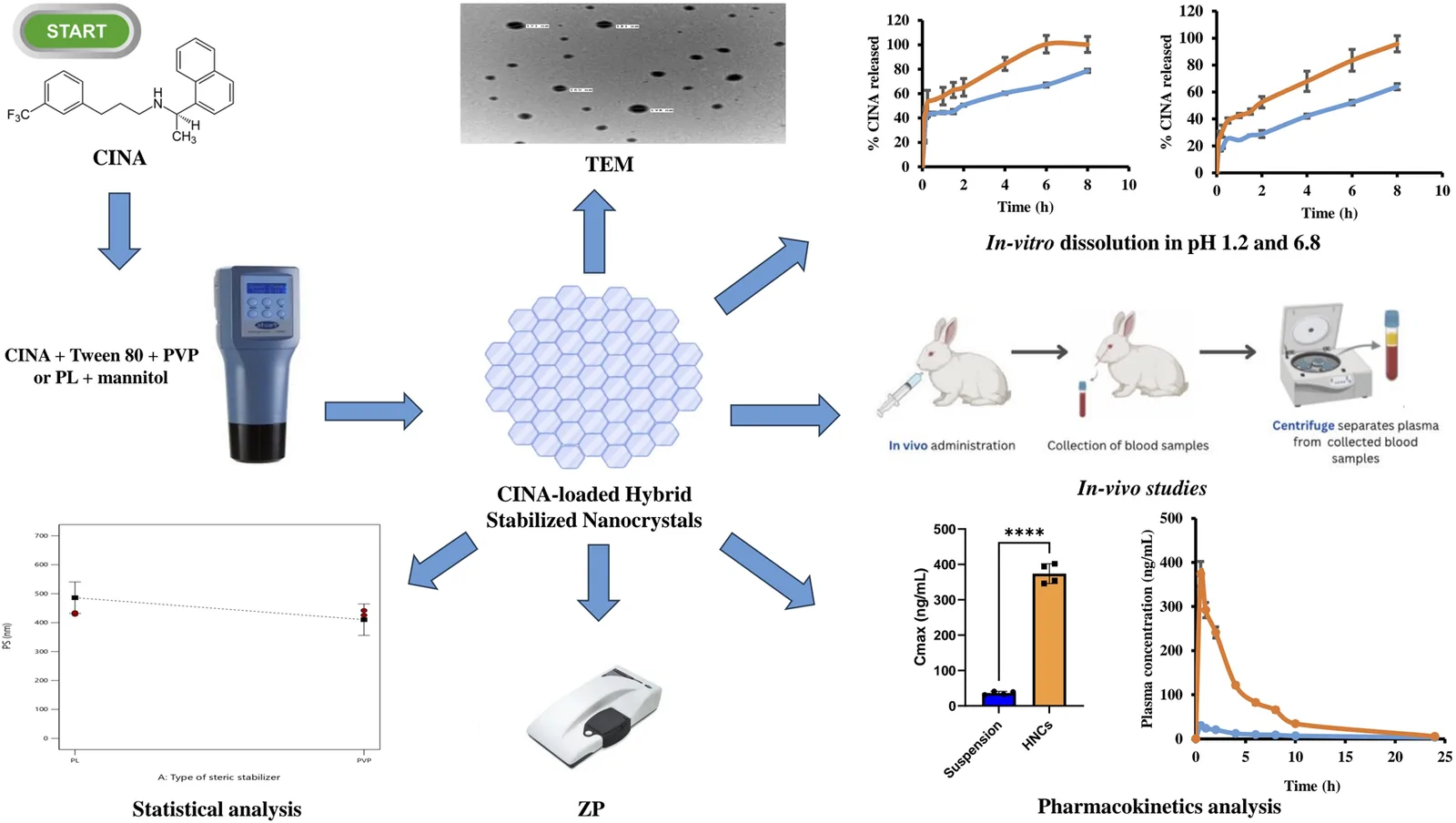

## Highlights

- Hybrid-stabilized nanocrystals of cinacalcet (CINA) were developed using steric (PVP K90 or Pluronic® F-127) and dispersing (Tween® 80) stabilizers to enhance solubility and stability.
- The optimum formula achieved a particle size (PS) of 193.44 nm, a poly-dispersity index (PDI) of 0.34, and a saturated solubility (SS) of 2.04 mg/mL (1.8-fold increase over unprocessed CINA).
- Transmission electron microscopy (TEM) confirmed uniformly distributed nanocrystals. Furthermore, the optimum formula demonstrated an improved drug release in simulated gastric and intestinal media, alongside favorable zeta potential (ZP) and physical stability.
- *In vivo* pharmacokinetics revealed 10.36- and 7.76-fold increases in maximum plasma concentration (Cmax) and area under the plasma concentration–time curve (AUC), respectively, compared to conventional CINA suspension, confirming superior systemic exposure.

## 1 Introduction

Chronic kidney disease (CKD) is a major global health problem and is frequently accompanied by secondary hyperparathyroidism (SHPT), particularly in patients with advanced stages of the disease (Lv et al., 2019; Nagib et al., 2023). SHPT develops as a consequence of impaired phosphate excretion and reduced production of active vitamin D, leading to disturbances in calcium and phosphate homeostasis and a compensatory increase in parathyroid hormone (PTH) secretion (Yamada and Nakano, 2023; Pazianas and Miller, 2021). Persistent elevation of PTH is associated with bone disorders, vascular calcification, and an increased risk of cardiovascular complications, highlighting the importance of effective long-term management (Cannata-Andia et al., 2011). Current therapeutic strategies for SHPT include vitamin D analogs, phosphate binders, and calcimimetics, which reduce PTH secretion by enhancing the sensitivity of the calcium-sensing receptor (CaSR) (Palumbo et al., 2021; Rastogi et al., 2021).

Cinacalcet (CINA), a second-generation calcimimetic, acts as a positive allosteric modulator of the CaSR on parathyroid chief cells, the principal locus of PTH biosynthesis and secretion, thereby stabilizing the receptor’s active conformation and suppressing PTH release in a calcium-dependent manner (Goto et al., 2024). CaSR detects fluctuations in extracellular calcium concentration and modulates PTH secretion accordingly (Nagano, 2006). Through allosteric modulation of the CaSR, CINA enhances the receptor’s sensitivity to extracellular calcium, thereby reducing PTH secretion and subsequently decreasing serum calcium and phosphate concentrations (Li et al., 2023). CINA is commercially available in 30, 60, and 90 mg tablet strengths. The poor aqueous solubility (∼9.2 × 10^−2^ mg/L) and high lipophilicity (LogP ≈ 6.5) of CINA limit its dissolution in the gastrointestinal tract and impede intestinal absorption, resulting in low oral bioavailability, reported to range from 20% to 25% (Padhi and Harris, 2009). This low solubility is primarily attributed to its lipophilic chemical structure, including an ethoxy-substituted benzene ring, and the stability of its crystalline lattice, which requires substantial energy to disrupt intermolecular interactions (Padhi et al., 2008). Consequently, CINA is classified as a Biopharmaceutics Classification System (BCS) Class IV compound, owing to its low solubility and poor permeability (Shree et al., 2023). Clinically, the oral bioavailability of CINA increases when administered with food, particularly a high-fat meal. This effect is primarily attributed to enhanced luminal solubilization by bile salts and mixed micelles, together with delayed gastric emptying, which prolongs drug residence in the acidic gastric environment and promotes dissolution before intestinal absorption.

Various formulation strategies have been explored to improve the oral delivery of CINA, including nanoemulsions, self-microemulsifying drug delivery systems (SMEDDSs), and lyophilized solid lipid nanoparticles (SLNs). Nanoemulsion-based systems have demonstrated improved drug dissolution and achieved a relative bioavailability of approximately 66.5%. Similarly, SMEDDSs have enhanced the solubility and oral bioavailability of CINA while minimizing the food effect through the formation of fine oil-in-water microemulsions upon dilution. In addition, lyophilized SLNs prepared by hot homogenization followed by ultrasonication increased the oral bioavailability by approximately two-fold compared with an aqueous suspension of the pure drug (Wang et al., 2022; Cao et al., 2018; Routray et al., 2020). Despite these promising outcomes, each delivery platform possesses inherent limitations. Lipid-based systems generally require relatively high concentrations of surfactants and co-solvents, which may raise concerns regarding gastrointestinal irritation and long-term safety. Moreover, they are susceptible to thermodynamic instability, drug leakage during storage, and drug precipitation upon dilution in gastrointestinal fluids, potentially compromising the *in vivo* performance. Likewise, SLNs often suffer from drug stability during storage. Previously, CINA nanocrystals prepared using a precipitation–ultrasonication method demonstrated enhanced dissolution and improved oral bioavailability, achieving approximately 1.90-fold and 1.64-fold increases in maximum plasma concentration (Cmax) and area under the plasma concentration–time curve (AUC), respectively, compared with the commercial product. Despite these encouraging findings, the formulation achieved a relatively larger particle size (∼244 nm). In contrast, the present study employed a hybrid stabilization strategy combining steric and dispersing stabilizers to produce smaller nanocrystals with improved physicochemical characteristics and enhanced oral pharmacokinetic performance (Xu et al., 2019). Consequently, improving the oral absorption of BCS Class IV drugs such as CINA remains a significant challenge as enhanced dissolution alone is insufficient to achieve optimal bioavailability (Bhalani et al., 2022). Therefore, developing a formulation that combines efficient particle size reduction with robust physical stabilization and sustained drug dispersion is highly desirable. Nanosystems have emerged as a versatile and powerful platform for improving the delivery and therapeutic performance of poorly soluble and poorly permeable drugs (Shaheen et al., 2026). By reducing particle size to the nanoscale, these systems increase surface area, enhance dissolution rates, and can modulate drug absorption across biological barriers (El-Dessouki et al., 2026). Beyond solubility enhancement, nanosystems allow precise control over particle stability, surface charge, and drug release kinetics, making them particularly suitable for oral delivery applications (Elmahboub et al., 2025; Senousy et al., 2026). Their tunable physicochemical properties, combined with the ability to integrate multiple stabilizers or functional excipients, offer a mechanistically rational approach to overcome the biopharmaceutical limitations of challenging drug candidates (Sedeek et al., 2026; Ahmed et al., 2026a). In this context, hybrid-stabilized nanocrystals (HNCs) represent a promising alternative by integrating the advantages of nanocrystal technology with complementary polymeric and surfactant stabilizers, providing synergistic steric and electrosteric stabilization to enhance dissolution, maintain colloidal stability, and improve oral drug absorption.

Nanocrystals are carrier-free submicron colloidal drug delivery systems with an average particle size typically ranging from 10 to 800 nm (Gigliobianco et al., 2018). Their primary objective is to enhance the oral bioavailability of poorly water-soluble drugs by markedly increasing the surface area-to-volume ratio, thereby increasing both solubility and dissolution rate (Liu et al., 2021). Commercial success has been achieved for several orally administered water-insoluble drugs, including Sirolimus (Rapamune®) and Fenofibrate (Tricor®) (Kong et al., 2019; Kevadiya et al., 2018). Nanocrystals are typically prepared via either top-down or bottom-up approaches, each offering distinct mechanical advantages. Top-down techniques mechanically reduce larger particles to the nanometer scale by employing some techniques such as high-pressure or high-speed homogenization, media milling, or ultrasonication, thereby achieving precise control over particle size distribution and enhancing dissolution (Van Eerdenbrugh et al., 2008). In contrast, bottom-up methods rely on controlled nucleation and crystal growth from drug solutions, including precipitation or supercritical fluid techniques, which allow tailoring of particle morphology and crystallinity (Ding et al., 2024). In addition, some hybrid approaches, including precipitation followed by high-pressure homogenization or ultrasonication and solution-grinding, combine the advantages of both strategies to further optimize nanocrystal properties and oral bioavailability. Among these methods, high-speed homogenization has been considered an attractive top-down method for producing nanocrystals with controlled particle size and long-term stability. During the process, the dispersion rapidly passes through a narrow gap between a high-speed rotating rotor and a stationary stator, exposing it to intense mechanical forces, including high-speed shearing, high-frequency oscillation, cavitation, and convection effects. These forces efficiently break down large particles into smaller particles, enabling rapid particle size reduction while maintaining a relatively simple setup, efficient energy input, and reproducible production of uniform nanocrystals suitable for oral administration (Zhou et al., 2019).

Concurrently, this method generates high-energy surfaces prone to aggregation and crystal growth. Stabilizers are, therefore, indispensable for controlling nanocrystal size and maintaining dispersion stability. Steric stabilizers, such as polymeric agents including polyvinylpyrrolidone K90 (PVP K90) and Pluronic® F-127, adsorb onto the nanocrystal surface to form a hydrated polymeric corona that provides steric hindrance, lowers surface free energy, and suppresses aggregation and Ostwald ripening (Phan and MacKay, 2024). In parallel, dispersing stabilizers, typically low-molecular-weight surfactants such as Tween® 80, rapidly reduce interfacial tension and improve wetting of the drug surface, thereby facilitating efficient particle breakage and uniform energy distribution during homogenization (Hansen and Kleinebudde, 2022). The synergistic integration of steric and dispersing stabilizers enables rapid surface coverage of freshly generated nanocrystals under extreme processing conditions, resulting in reduced particle size, narrower size distributions, and enhanced colloidal stability. This hybrid stabilization strategy is thus pivotal for achieving reproducible nanocrystal production and preserving the physicochemical integrity of the nanosuspension during and after high-pressure homogenization.

PVP K90 is a water-soluble polymer synthesized through N-vinylpyrrolidone polymerization. It is inert, non-toxic, temperature-resistant, pH-stable, biocompatible, and biodegradable (Kurakula and Rao, 2020). Pluronic F-127 is an amphiphilic triblock copolymer composed of two hydrophilic polyethylene oxide (PEO) chains linked by a central hydrophobic polypropylene oxide (PPO) segment. Its low molecular weight and high PEO content promote micelle formation and gelation at critical micelle concentrations and temperatures, enhancing solubilization of active substances (Khaliq et al., 2023). Tween® 80 (polysorbate 80) is widely used as a dispersing stabilizer in nanocrystal formulations due to its strong surface activity and low molecular weight. Tween® 80 rapidly adsorbs onto freshly generated drug surfaces, effectively reducing interfacial tension and enhancing wettability, which facilitates efficient particle breakage and uniform size reduction (Ahmed et al., 2026b).

In this study, HNCs of CINA were prepared via high-speed homogenization technique, aiming to develop an oral CINA formulation in a nano-sized range with optimum safety and efficacy. A 2^1^ × 3^2^ mixed-level factorial design was developed to optimize the formulation, investigating the effects of A: type of steric stabilizers, B: total stabilizer concentration (mg/mL), and C: dispersing stabilizer contribution (% w/w). The selected dependent responses were Y1: particle size (PS), Y2: poly-dispersity index (PDI), and Y3: drug saturated solubility in water. Moreover, *in vitro* characterization and *in vivo* pharmacokinetic studies were conducted to evaluate the solubility and oral bioavailability of the optimized CINA HNCs.

## 2 Materials and methods

### 2.1 Materials

CINA was kindly supplied by Global Napi Pharmaceuticals (Cairo, Egypt). PVP K90 was obtained from Loba Chemie Pvt. Ltd. (Mumbai, India), while Pluronic® F-127 was purchased from Sigma-Aldrich (St. Louis, MO, United States). Tween® 80 was sourced from Piochem Laboratory Chemicals (Giza, Egypt). Mannitol was provided by TopChem Pharmaceuticals (Sligo, Ireland). All additional reagents and solvents utilized throughout the study were of analytical grade and used as received without further purification.

### 2.2 Experimental design for optimizing HNCs of CINA

A 2^1^ × 3^2^ mixed-level factorial design was adopted to systematically investigate the influence of formulation variables on the characteristics of HNCs. The independent variables were defined as follows: A: type of steric stabilizers, B: total stabilizer concentration (mg/mL), and C: dispersing stabilizer contribution (% w/w). The selected dependent responses were as follows: Y1: PS, Y2: PDI, and Y3: drug saturated solubility in water. The experimental matrix was constructed using Design-Expert® software (Version 13.0.0, Stat-Ease Inc., Minneapolis, MN, United States), generating a total of 18 proposed HNC formulations (Eldeeb et al., 2026). Each formulation was prepared and evaluated in triplicate (n = 3) to enable estimation of pure experimental error and assessment of model adequacy. Factor A was evaluated at two categorical levels (Pluronic® F-127 or PVP K90), while factors B and C were assessed at three quantitative levels each. Total stabilizer concentration (B) was studied at 4.5, 6.0, and 7.5 mg/mL, whereas dispersing stabilizer contribution (C) was investigated at 20%, 40%, and 60% w/w. The selected ranges for factors B and C were selected based on preliminary screening studies. The complete experimental layout is presented in Table 1. The measured responses (Y1, Y2, and Y3) were statistically analyzed using multiple regression modeling to establish the relationship between the independent variables and formulation performance. Model adequacy and predictive capability were evaluated based on adjusted R^2^, predicted R^2^, adequate precision, and corresponding P-values to identify the most statistically appropriate model for each response (Ahmed et al., 2026c; El Hassab et al., 2025). The prepared formulations and their experimentally observed response values are summarized in Table 2.

**TABLE 1:** 2^1^ × 3^2^ mixed factorial design layout: selected factors, studied responses, and desirability goals.

Factor (independent variable)	Level		
	−1	0	+1
A: Type of steric stabilizers	PL	—	PVP
B: Total stabilizer concentration (mg/mL)	4.5	6	7.5
C: Dispersing stabilizer contribution (%w/w)	20	40	60

Response (dependent variable)	Desirability constraints		
Y1: PS (nm)	Minimize		
Y2: PDI	Minimize		
Y3: SS (mg/mL)	Maximize		

Abbreviations: PDI, poly-dispersity index; PL, Pluronic® F-127; PVP, Poly-vinyl-pyrrolidone; PS, particle size; SS, saturated solubility.

**TABLE 2:** CINA hybrid-stabilized nanocrystal formulations prepared according to the 2^1^ × 3^2^ mixed factorial design (n = 3, mean ± SD).

Formula	Factors			Composition (mg/mL)		Response		
	Factor A: type of steric stabilizers	Factor B: total stabilizer concentration (mg/mL)	Factor C: dispersing stabilizer contribution (%w/w)	Steric stabilizer (PL or PVP)	Dispersing stabilizer (Tween 80)	PS (nm)	PDI	Saturated solubility (mg/mL)
F1	PL	4.5	20	3.6	0.9	498.60 ± 2.40	0.25 ± 0.03	1.56 ± 0.04
F2	PL	4.5	40	2.7	1.8	482.40 ± 6.97	0.31 ± 0.01	1.26 ± 0.05
F3	PL	4.5	60	1.8	2.7	311.95 ± 5.63	0.54 ± 0.03	1.63 ± 0.02
F4	PL	6	20	4.8	1.2	437.15 ± 8.61	0.47 ± 0.04	1.65 ± 0.03
F5	PL	6	40	3.6	2.4	637.55 ± 9.49	0.50 ± 0.03	1.43 ± 0.01
F6	PL	6	60	2.4	3.6	454.85 ± 8.48	0.41 ± 0.01	1.46 ± 0.05
F7	PL	7.5	20	6	1.5	579.15 ± 4.60	0.43 ± 0.04	1.51 ± 0.02
F8	PL	7.5	40	4.5	3	414.50 ± 7.18	0.47 ± 0.02	1.14 ± 0.02
F9	PL	7.5	60	3	4.5	431.50 ± 4.10	0.46 ± 0.02	1.20 ± 0.07
F10	PVP	4.5	20	3.6	0.9	168.15 ± 2.83	0.41 ± 0.02	2.01 ± 0.01
F11	PVP	4.5	40	2.7	1.8	115.05 ± 6.62	0.50 ± 0.01	1.45 ± 0.04
F12	PVP	4.5	60	1.8	2.7	209.30 ± 3.11	0.35 ± 0.02	1.47 ± 0.03
F13	PVP	6	20	4.8	1.2	96.77 ± 7.81	0.34 ± 0.03	1.61 ± 0.03
F14	PVP	6	40	3.6	2.4	239.65 ± 5.67	0.28 ± 0.01	1.66 ± 0.03
F15	PVP	6	60	2.4	3.6	206.15 ± 3.18	0.35 ± 0.01	2.03 ± 0.08
F16	PVP	7.5	20	6	1.5	73.12 ± 6.48	0.43 ± 0.03	1.64 ± 0.05
F17	PVP	7.5	40	4.5	3	383.20 ± 5.37	0.38 ± 0.01	1.76 ± 0.05
F18	PVP	7.5	60	3	4.5	433.80 ± 8.46	0.38 ± 0.02	1.58 ± 0.04

Abbreviations: CINA, cinacalcet; PDI, poly-dispersity index; PL, Pluronic® F-127; PVP, Poly-vinyl-pyrrolidone; PS, particle size; SS, saturated solubility.

NB: 30 mg of CINA was dispersed in 20 mL of distilled water containing the designated stabilizers.

### 2.3 Preparation of CINA HNCs

HNCs of CINA were produced using a high-speed homogenization approach. In brief, 30 mg of CINA was dispersed in 20 mL of distilled water containing the designated steric stabilizer (Pluronic® F-127 or PVP K90) and dispersing stabilizer (Tween® 80) according to the compositions outlined in Table 1. The mixture was initially magnetically stirred (Daihan MSH-20A hotplate magnetic stirrer, South Korea) at 500 rpm for 5 min to ensure complete dissolution and uniform distribution of the formulation components. Subsequently, particle size reduction and nanocrystal formation were achieved via high-shear homogenization (Stuart SHM3 High-Shear Homogenizer, United Kingdom) operated at 20,000 rpm for 30 min (Zhou et al., 2022). The homogenization speed and duration were selected based on preliminary single-factor optimization trials. This process facilitated the generation of nanoscale drug crystals stabilized using the hybrid surfactant system. Following homogenization, mannitol (0.8 g, corresponding to 4% w/v of the total suspension volume) was incorporated as a cryoprotective agent. This concentration was selected based on previous reports demonstrating its effectiveness in preserving the physicochemical stability of nanosuspensions during freeze-drying. The dispersion was then gently stirred at 300 rpm for an additional 5 min to ensure homogeneous distribution of the cryoprotectant (Khan et al., 2024). The prepared dispersions were then frozen at −80 °C for 24 h to promote complete solidification prior to drying. Freeze-drying was subsequently carried out (Buchi L-200 lyophilizer, Switzerland) for 72 h at −20 °C under a vacuum of 0.500 mbar, yielding dry nanocrystal powder suitable for further characterization (Ahmed et al., 2021).

### 2.4 *In vitro* characterization of the HNCs of CINA

To ensure strict experimental reproducibility and eliminate operator variability, we have standardized the reconstitution procedure for all lyophilized samples prior to characterization. The procedure involves adding a fixed volume (10 mL) of Milli-Q water to the freeze-dried cake, followed by gentle manual inversion for 30 s and a brief low-power bath sonication (30 s at 25 °C) to guarantee uniform dispersion without applying excessive shear force that could artificially disrupt primary particles.

#### 2.4.1 Particle size and poly-dispersity index analysis

The mean PS and PDI of the prepared formulations were determined using a Zetasizer Nano ZS instrument (Malvern Instruments, United Kingdom) based on dynamic light scattering (DLS) technology (Albash et al., 2025). To ensure accurate measurement and minimize multiple scattering effects, the lyophilized HNCs were appropriately diluted with distilled water at a ratio of 1:100 prior to analysis (Saher et al., 2019). The 1:100 dilution was selected to operate within the linear detection range of the instrument, preventing multiple scattering artifacts while maintaining sufficient scattered light intensity (Elmahboub et al., 2026). For all reported measurements, the mean count rate consistently ranged between 200 and 350 kilocounts per second. Measurements were conducted at 25 °C using a fixed backscattering angle of 173°, allowing reliable detection of nanoscale particle distributions (Fah et al., 2026). Each sample was analyzed in triplicate to ensure reproducibility, and the obtained data were expressed as mean values. The recorded PS and PDI results were systematically compiled for subsequent statistical evaluation (Patil et al., 2021).

#### 2.4.2 Saturated solubility determination

A SS study was performed for both CINA HNCs and pure CINA. An excess amount of each sample was dispersed in 5 mL of distilled water and maintained in a thermostatically controlled water bath shaker (Daihan Scientific, South Korea) at 37.5 °C and 200 strokes per minute for 72 h to achieve equilibrium solubility (Yassin and Khalifa, 2022). Upon completion of the 72-h shaking period, the mixtures were filtered through a 0.45-µm filter paper to remove undissolved residues. The resulting saturated supernatants were appropriately diluted with distilled water, and CINA concentrations were quantified spectrophotometrically using a Shimadzu UV-1900 UV-Vis spectrophotometer (Kyoto, Japan) at a predetermined wavelength of 313.6 nm. All measurements were conducted in triplicate, and mean values were calculated and documented (Xu et al., 2019).

### 2.5 Identification of the optimized CINA HNC formula

The optimal HNC formulation was determined using Design-Expert® software (Version 13.0.0, Stat-Ease Inc., Minneapolis, MN, United States) through numerical optimization based on a predefined desirability function (Hussein et al., 2024). The optimization criteria were established to minimize PS and PDI while maximizing SS, thereby ensuring enhanced physicochemical performance. These response goals were collectively incorporated into a composite desirability index to identify the most favorable formulation within the experimental design space. The validity of the optimization process was further confirmed by calculating the percentage deviation between the predicted and experimentally observed responses, which was determined using Equation 1 (Ahmed et al., 2026d):

$$\%\text{ Deviation}=\left(\left|\text{Predicted value }-\text{ Observed value}\right|/\left(\text{Observed value}\right)\right)\times 100.$$ (1)

To confirm minimal drug loss during the nano-formulation process and ensure uniform drug loading, the drug content (DC%) of the optimized hybrid nanocrystals was determined using UV–visible spectrophotometry. The absorbance was measured at the predetermined wavelength (λmax). DC was measured using Equation 2:

$$\text{DC}\%=\frac{actual\;drug\;amount}{added\;drug\;amount}\times 100.$$ (2)

### 2.6 Further characterization of the optimized CINA HNCs

#### 2.6.1 *In vitro* dissolution study

The *in vitro* release profile of the optimized HNCs was investigated compared with pure CINA using a dialysis membrane diffusion method (Farag et al., 2022). Dissolution testing was performed in two physiologically relevant media to simulate gastric and intestinal environments: 0.1 N hydrochloric acid and phosphate buffer (pH 6.8), each with a total volume of 100 mL. Accurately weighed amounts (15 mg) of pure CINA and the lyophilized optimized HNC formulation were separately dispersed in 2 mL of distilled water and transferred into pre-soaked dialysis membranes with a molecular weight cut-off (MWCO) of 14,000 Da, which were securely sealed at both ends. The loaded dialysis bags were then placed in amber-colored glass containers filled with the designated dissolution medium to protect the samples from potential light exposure (Ahmed et al., 2025a). Sink conditions were maintained throughout the dissolution study as the receptor medium volume substantially exceeded the minimum volume required to dissolve the applied 15 mg dose based on the experimentally determined saturation solubility of CINA. According to USP recommendations, sink conditions are achieved when the dissolution-medium volume is at least three times the volume required to saturate the medium. In addition, control experiments using molecularly dissolved CINA confirmed rapid membrane transport within 1 h, indicating that the 14 kDa dialysis membrane was not rate-limiting for drug diffusion. The containers were then maintained in a thermostatically controlled shaking water bath (Daihan Scientific, South Korea) at 37 °C ± 0.5 °C with continuous agitation at 200 strokes per minute to ensure uniform mixing and diffusion (Weng et al., 2020). At specified time intervals (0.25, 0.5, 1, 2, 4, 6, and 8 h), 5 mL samples were withdrawn and immediately replaced with an equivalent volume of fresh pre-warmed medium to preserve constant volume and sink conditions (Ahmed et al., 2025b). The collected samples were filtered when necessary and analyzed using a UV–visible spectrophotometer at 313.6 nm. Drug concentrations were calculated from a previously constructed calibration curve, and the cumulative percentage of drug released was determined using Equation 3 (Polli, 2022):

$$Qn=\frac{Cn\;.\;Vr+\sum_{i=1}^{n-1}Ci\;.\;VS}{initial\;drug\;content}\times 100,$$ (3)

where

- Qn: cumulative percentage of the drug released at the nth sampling time.
- Cn: drug concentration in the receiver medium at the nth sampling time.
- Vr: total volume of the receiver medium.
- Vs: volume of each sample withdrawn for analysis.
-
$\sum_{i=1}^{n-1}Ci$ : sum of the drug concentrations measured in all previously withdrawn samples

To elucidate the mechanism governing drug release from the optimized hybrid nanocrystals, the *in vitro* release data were fitted to different mathematical kinetic models, including the zero-order, first-order, Higuchi, and Korsmeyer–Peppas models. Linear regression analysis was performed for each model, and the corresponding coefficient of determination (R^2^) was calculated. The kinetic model exhibiting the highest R^2^ value was considered to best describe the release behavior. Furthermore, dissolution efficiency (DE) was calculated using Khan and Rhodes’ equation (Equation 4) (Simionato et al., 2018):

$$\text{DE}=\frac{\int_{0}^{t}Y\cdot dt}{Y_{100}\cdot t}\times 100,$$ (4)

where

- Y is the percentage of the drug dissolved at any given time t.
-
$\int_{0}^{t}Y\cdot dt$ represents the AUC from time zero up to time t.
- 100 ⋅ t represents the total potential area if 100% of the drug had dissolved instantaneously at t = 0 and remained at 100% until time t

#### 2.6.2 Saturated solubility in simulated gastrointestinal media

To evaluate the solubility behavior of the optimized formula under physiologically relevant conditions, the saturated solubility was determined in simulated gastric fluid (SGF, 0.1 N HCl, pH 1.2) and simulated intestinal fluid (SIF, phosphate buffer, pH 6.8). An excess amount of the optimized hybrid nanocrystals was added separately to each dissolution medium and incubated at 37 °C ± 0.5 °C for 72 h under continuous shaking to ensure equilibrium. The resulting suspensions were centrifuged, and the supernatants were carefully collected and filtered through a 0.45-μm membrane filter. The concentration of dissolved cinacalcet was quantified using a validated UV–visible spectrophotometric method after appropriate dilution. All experiments were carried out in triplicate, and the results were expressed as mean ± standard deviation (SD).

#### 2.6.3 Transmission electron microscopy (TEM) analysis

Morphology of the optimized CINA HNC formulation was examined using transmission electron microscopy (TEM) (Ahmed et al., 2025c). A defined amount of the lyophilized formulation was diluted with distilled water (1:0) to prepare the TEM sample. A drop of the diluted sample was placed onto a carbon-coated copper grid and negatively stained with phosphotungstic acid (Elgendy et al., 2024). The grid was then allowed to air-dry prior to examination under the TEM at different magnifications (Fahmy et al., 2025).

#### 2.6.4 Zeta potential measurement

The surface charge of the optimized HNC formulation was evaluated through zeta potential (ZP) measurements (Ahmed et al., 2026e). Prior to analysis, the optimized formula was appropriately diluted with deionized water to minimize particle–particle interactions and reduce potential scattering artifacts, ensuring accurate readings. Measurements were performed using a Nano Zetasizer (Model ZEN3600, Malvern Instruments Ltd., United Kingdom), which determines zeta potential by assessing the electrophoretic mobility of the nanoparticles under an applied electric field (Soroushnia et al., 2021). The obtained values provide an indication of the electrostatic stability and potential aggregation propensity of the formulation (El-Naggar et al., 2026). ZP measurements were conducted under identical experimental conditions to ensure consistency and improve the reproducibility of the reported results.

#### 2.6.5 FTIR

Fourier-transform infrared (FTIR) spectroscopy was performed to evaluate the chemical structure of cinacalcet before and after the preparation of the hybrid nanocrystals and to detect any possible drug–excipient interactions. The FTIR spectra of raw cinacalcet and the optimized hybrid nanocrystals were recorded using a Bruker FTIR spectrophotometer (Model 22, Coventry, United Kingdom) (Ahmed et al., 2026g). Before analysis, the samples were thoroughly dried, mixed with potassium bromide (KBr), and compressed into transparent pellets. The spectra were collected over a wavenumber range of 4,000–500 cm^−1^ at 25 °C. The obtained spectra were compared by examining the characteristic absorption bands to identify any changes in peak position, intensity, or the appearance of new peaks, which may indicate molecular interactions or structural changes during hybrid nanocrystal preparation (Ahmed et al., 2026b).

#### 2.6.6 X-ray powder diffraction (XRPD)

The crystalline characteristics of raw CINA and the optimized HNC formulation were evaluated using an X-ray diffractometer (XD-610, Shimadzu, Kyoto, Japan). The samples were analyzed at a voltage of 45 kV and a current of 40 mA. Diffraction patterns were recorded over a 2θ range of 10°–80°, using a scanning rate of 2°/min and a step size of 0.02° (El Assasy et al., 2019).

#### 2.6.7 Stability assessment

The stability of the optimized HNC formulation was evaluated under both refrigerated and accelerated storage conditions. For the refrigerated study, samples were stored at 5 °C ± 3 °C for 90 days to assess the ability of the nanocrystals to maintain their physicochemical properties during storage (Sayed et al., 2021a). In addition, accelerated stability testing was performed in accordance with ICH climatic Zone IV guidelines by storing the packaged formulation at 40 °C ± 2 °C/75% ± 5% relative humidity (RH) for 1 month. For both studies, the formulation was filled into Type I glass vials, sealed with chlorobutyl rubber stoppers, and crimped with aluminum flip-off seals to provide protection against moisture and light (Teama et al., 2025). Critical quality attributes, including PS, PDI, ZP, and SS, were re-measured at the end of the storage period and statistically compared to the initial values using Student’s t-test to detect any significant deviations (Ahmed et al., 2025d). Additionally, the formulations were subjected to visual inspection to qualitatively assess physical stability, monitoring for potential signs of aggregation, sedimentation, or precipitation (Al-Mahallawi et al., 2017).

### 2.7 *In vivo* studies

#### 2.7.1 Animals

The optimized HNC formulation was selected for a parallel *in vivo* pharmacokinetic investigation to evaluate its potential to enhance the oral bioavailability of CINA. Eight healthy male rabbits (2.0 ± 0.5 kg) were procured from the Animal House, Faculty of Pharmacy, Cairo University, Egypt, and maintained under standardized laboratory conditions, including controlled temperature and light cycles, with unrestricted access to water and a standard fiber-enriched diet throughout the experimental period. All animal handling and experimental procedures were conducted in strict accordance with ethical standards and were approved by the Research Ethics Committee of the Faculty of Pharmacy, New Giza University, Giza, Egypt (Approval No. PI-0092). Animals were monitored closely to ensure welfare, and all interventions were designed to minimize stress and discomfort, fully adhering to the ARRIVE guidelines. The choice of n = 4 per group (8 rabbits in total) was designed for a pilot screening study intended to assess the enhancement of oral bioavailability by HNCs versus the pure drug. In accordance with the 3Rs principles (reduce, reuse, and recycle), animal numbers were kept to the minimum necessary for an initial proof-of-concept evaluation. All animals underwent a mandatory 7-day acclimatization period prior to study initiation.

#### 2.7.2 Pharmacokinetics study

Under fed conditions, the animals were randomly divided into two groups (n = 4), designated as Group A and Group B. Group A received the optimized CINA HNC formulation orally as an aqueous dispersion at a dose equivalent to 8 mg, while Group B received an oral suspension of the pure drug at the same dose. A parallel study design was chosen to eliminate potential carryover effects and avoid extended washout periods that could introduce intra-subject physiologic variability associated with animal growth and weight gain over time. Blood samples (0.5 mL) were collected via the marginal ear vein using sterile, heparinized syringes at predetermined time points (0.5, 1, 2, 4, 6, 8, 10, and 24 h), centrifuged at 4,800 rpm for 15 min using an 82 Electronic Laboratory Medical Centrifuge (Changzhou, Jiangsu, China), and the plasma was separated for analysis. Plasma concentrations of CINA were quantified using linagliptin as the internal standard (IS). In brief, 0.5 mL of plasma was spiked with 50 μL of linagliptin solution (2 μg/mL) and subjected to liquid–liquid extraction using 4 mL of ethyl acetate. The mixture was vortex-mixed for 2 min and centrifuged at 4,000 rpm for 10 min. The organic layer was carefully collected and evaporated to dryness under reduced pressure. The resulting residue was reconstituted in 500 μL of acetonitrile, vortexed thoroughly, and filtered through a 0.22-μm syringe filter prior to LC–MS/MS analysis.

Chromatographic separation was performed using an Agilent Eclipse Plus C18 column (50 mm × 4.6 mm, 5 μm) at ambient temperature. The mobile phase, consisting of 2.5 mM ammonium formate in methanol, was delivered at a flow rate of 1.0 mL/min. Pharmacokinetic parameters, including the area under the plasma concentration–time curve (AUC_0–24_ h), Cmax, time to reach maximum plasma concentration (Tmax), terminal elimination rate constant (λz), relative bioavailability (F_rel_), and mean residence time (MRT) were calculated using Phoenix® software (version 8.5.2, Phoenix Technologies Ltd., Washington, United States) (Ragheb et al., 2026). Statistical analysis was performed using the independent samples t-test with GraphPad Prism software (version 9.6.1, San Diego, CA, United States), and differences were considered statistically significant at p < 0.05 (Mayer et al., 2014).

The LC–MS/MS method was validated in accordance with the FDA and EMA bioanalytical guidelines. Calibration curves exhibited excellent linearity over the concentration range of 2.8–460.0 ng/mL (r^2^ = 0.9999) using a 1/x^2^ weighted linear regression. The lower limit of quantification (LLOQ) was 2.8 ng/mL, while intra- and inter-day accuracy ranged from 94.2% to 106.8% with precision (RSD) below 8.5%, meeting the recommended acceptance criteria. Linagliptin was selected as the internal standard due to its reproducible chromatographic and mass spectrometric performance, and no interference was observed between the analyte and internal standard, confirming the selectivity and reliability of the analytical method.

### 2.8 Statistical analysis

The experimental data are expressed as the mean ± SD of triplicate measurements (Bazaz et al., 2021). The data were analyzed using one-way analysis of variance (ANOVA) implemented in Design Expert® software (version 13.0.0, Stat-Ease Inc., Minneapolis, MN, United States), with significance considered at P < 0.05 (Daralnakhla et al., 2021; Saher et al., 2023).

## 3 Results and discussion

### 3.1 Factorial design

The statistical indices presented in Table 3 demonstrate the adequacy and predictive capability of the developed models. In particular, the comparison between the adjusted R^2^ and predicted R^2^ values showed that their differences remained below the acceptable threshold of 0.20 for the evaluated responses, indicating good agreement between the models’ explanatory and predictive performance (AbouHussein et al., 2026). Furthermore, the adequate precision values exceeded the recommended minimum value of 4, indicating an adequate signal-to-noise ratio and confirming that the developed models provide an appropriate basis for navigating the design space and optimization (Tawfik et al., 2025). Collectively, these parameters substantiate the reliability of the experimental design in elucidating the effects of formulation variables on the HNC fabrication and affirm that the predicted optimal conditions are both reproducible and statistically significant (Habib et al., 2018; Ahmed et al., 2023).

**TABLE 3:** Summary of model statistics for the measured responses.

Response	R^2^	Adjusted R^2^	Predicated R^2^	Adequate precision	Significant factors
PS (nm)	0.8152	0.7769	0.7351	12.54	A and B
PDI	0.8691	0.7590	0.6581	10.58	A
SS (mg/mL)	0.9664	0.9411	0.8967	22.42	A and C

Abbreviations: PDI, poly-dispersity index; PS, particle size; SS, saturated solubility.

#### 3.1.1 Particle size and PDI analysis

PS represents a fundamental attribute governing the performance of nanocrystal-based drug delivery systems as it directly influences dissolution behavior, colloidal stability, and ultimately oral bioavailability (Ragheb et al., 2026). Reduction in particle size to the nanometer scale significantly increases the specific surface area available for dissolution, which accelerates drug release and improves apparent solubility, thereby facilitating enhanced gastrointestinal absorption (Safwat et al., 2017). In the present factorial design, the PS of the prepared HNCs varied markedly across the 18 experimental runs, ranging from 73.12 ± 6.48 nm to 637.55 ± 43.49 nm. In parallel, the PDI serves as an indicator of the uniformity of particle size distribution within the nanosystem. Lower PDI values denote a more homogeneous and monodisperse population of particles, whereas higher values indicate broader size variability (Ahmed et al., 2026c). Such heterogeneity may influence dissolution behavior and physical stability as smaller particles tend to dissolve rapidly, while larger particles dissolve more gradually, potentially resulting in inconsistent solubility profiles. Within the experimental design, PDI values ranged from 0.249 ± 0.030 to 0.536 ± 0.033 across the developed formulations.

**FIGURE 1:**
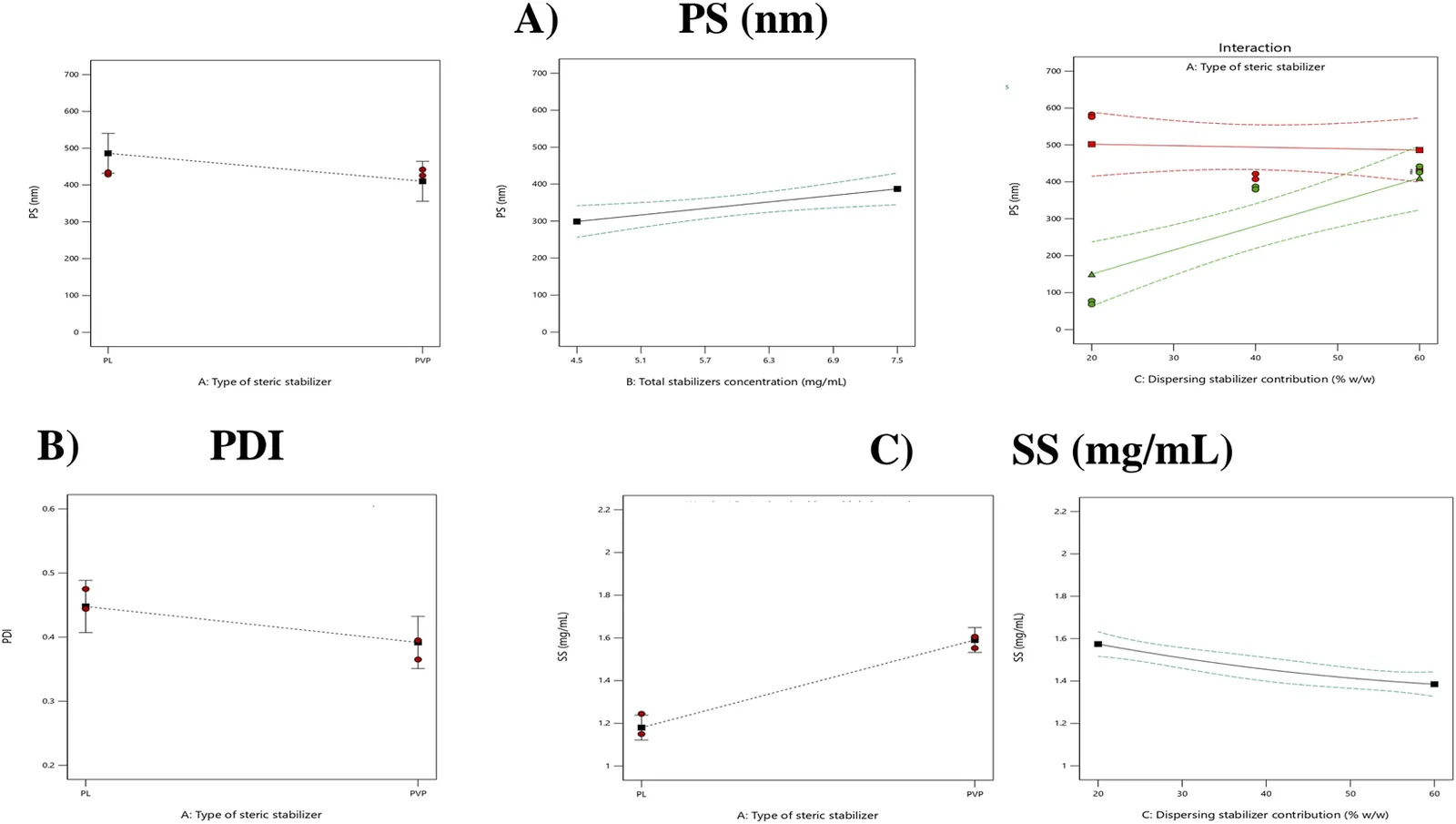
Response plots for the effect of type of steric stabilizers (Factor A), total stabilizer concentration (Factor B), and dispersing stabilizer contribution (Factor C) on **(A)** PS, **(B)** PDI, and **(C)** SS, highlighting the significant effects of the studied factors (*p* < 0.05).

Statistical evaluation revealed that Factor-A (type of steric stabilizers) exerted a highly significant influence on both PS and PDI (p < 0.0001), as illustrated in Figures 1A,B. Formulations stabilized with PVP K90 consistently produced smaller particle sizes and narrower size distributions than those containing Pluronic F127, indicating that the selected polymer markedly influenced nanocrystal formation during homogenization. This difference may be related to the distinct physicochemical properties of the two polymers, which can affect their ability to stabilize newly generated nanocrystals and limit particle growth. Similar trends have been reported in previous studies, where PVP-based stabilizers provided improved control over particle size and size distribution compared with other polymeric stabilizers (Ahmadi Tehrani et al., 2019; Dantas Lopes Dos Santos et al., 2021).

In addition, Factor B (total stabilizer concentration) exerted a significant positive effect on particle size (Figure 1A), with higher stabilizer concentrations producing larger particles (p < 0.05). This finding suggests that increasing the polymer content beyond the optimal level may reduce the efficiency of the homogenization process and increase the hydrodynamic diameter measured by dynamic light scattering. Moreover, The ANOVA revealed a significant interaction between the type of steric stabilizers (Factor A) and the dispersing stabilizer contribution (Factor C) on nanoparticle size ( p=0.0002 ). As depicted in Figure 1A, the effect of Tween 80 concentration was highly dependent on the choice of the primary polymer. For formulations using Pluronic F 127 (A1), particle size remained relatively invariant ( ∼500 nm ) across the entire range of dispersing stabilizer ratios ( 20%–60% w/w ). Conversely, formulations stabilized with PVP K 90 exhibited a pronounced increase in particle size, escalating from 150 nm at a 20% contribution to more than 400 nm at 60% . This distinct behavior can be rationalized by competitive adsorption dynamics at the particle interface. At lower dispersing stabilizer ratios ( 20% ), PVP K 90 forms a robust, high-molecular-weight steric barrier that effectively prevents aggregation. However, increasing the proportion of the small-molecule surfactant (Tween 80) to 60% likely leads to the competitive displacement of PVP from the nanoparticle surface, weakening steric protection and encouraging particle growth or aggregation. In contrast, the amphiphilic block copolymer structure of Pluronic F 127 maintains strong surface anchoring alongside Tween 80, rendering its steric stability insensitive to variations in dispersing stabilizer concentration. Consequently, pairing PVP K 90 with a lower dispersing stabilizer contribution ( 20% w/w ) was identified as the optimal condition for achieving minimal particle size.

#### 3.1.2 Saturated solubility

Saturated solubility refers to the maximum concentration of a drug that can dissolve in a solvent when equilibrium is established between the dissolved molecules and the excess undissolved drug at a constant temperature (Aghrbi et al., 2021). This parameter represents a key determinant governing dissolution performance, particularly for orally administered drugs where gastrointestinal transit time is limited. The oral absorption of a drug is strongly influenced by several physicochemical and biological factors, including aqueous solubility, dissolution rate, membrane permeability, efflux transporters, and hepatic first-pass metabolism (Zheng et al., 2022). In the present investigation, the saturated solubility of raw CINA and the prepared HNC formulations was determined in distilled water. The results demonstrated that the saturated solubility of the 18 developed formulations ranged from 1.20 ± 0.07 to 2.03 ± 0.08 mg/mL. Compared with the unprocessed drug (1.10 ± 0.02 mg/mL), all HNC formulations exhibited a noticeable improvement in aqueous solubility. This enhancement can be primarily attributed to the nanoscale size of the particles, which increases surface area and promotes improved drug–solvent interaction (Ahmed et al., 2026d).

Statistical analysis demonstrated that Factor A (type of steric stabilizers) had a highly significant effect on the saturated solubility of CINA (p < 0.0001) (Figure 1C). Formulations containing PVP K90 consistently exhibited higher saturated solubility than those prepared with Pluronic F127. This finding is consistent with the smaller particle sizes obtained with PVP K90, suggesting that the improved solubility was associated with more efficient particle size reduction and stabilization. Similar observations have been reported for PVP-stabilized nanocrystal systems, where improved particle size control was accompanied by enhanced apparent solubility. Furthermore, Factor-C [dispersing stabilizer contribution (%w/w)] showed a statistically significant negative impact on saturated solubility. Increasing the relative proportion of Tween 80 in PVP-stabilized systems resulted in a gradual decrease in the measured solubility of CINA. This behavior may be attributed to competitive adsorption of Tween 80 molecules onto the nanocrystal surface, which can partially interfere with the adsorption of PVP chains. Such competition may reduce the effectiveness of steric stabilization, potentially leading to partial particle growth and a subsequent decrease in the apparent solubility of the drug.

### 3.2 Optimization of CINA HNCs

Optimization was conducted with the objective of minimizing particle size and poly-dispersity index while maximizing saturated solubility. Using the desirability function approach, the formulation exhibiting the highest overall numerical desirability (0.741) was identified as the optimal HNCs. This optimized formulation contained a total stabilizer concentration of 5.73 mg/mL, employing PVP K90 as the steric stabilizer and Tween 80 at a relative percentage (60%). To validate the adequacy of the statistical model, the optimized formulation was prepared and experimentally evaluated. The experimental results (Table 4) revealed a close agreement between the predicted and experimental values (<5%) (Helal et al., 2024; Yousry et al., 2020). The mean drug content for the optimized formulation was determined to be 107.0% ± 2.3%. This value satisfies international pharmacopeial acceptance criteria (USP/Ph. Eur. acceptable limits of 90.0%–110.0% of the nominal label claim). The high drug content confirms minimal drug loss during the nano-formulation processing steps and demonstrates uniform drug distribution within the hybrid stabilizer matrix.

**TABLE 4:** The predicted and observed responses of the optimized formula.

Response	Y1	Y2	Y3
	PS (nm)	PDI	SS (mg/mL)
Observed value	193.44	0.34	2.04
Predicated value	201.85	0.33	1.98
% deviation (absolute)	4.35%	3.03%	2.94%

Abbreviations: PDI, poly-dispersity index; PS, particle size; SS, saturated solubility.

### 3.3 Further characterization of the optimized CINA HNCs

#### 3.3.1 *In vitro* dissolution study

**FIGURE 2:**
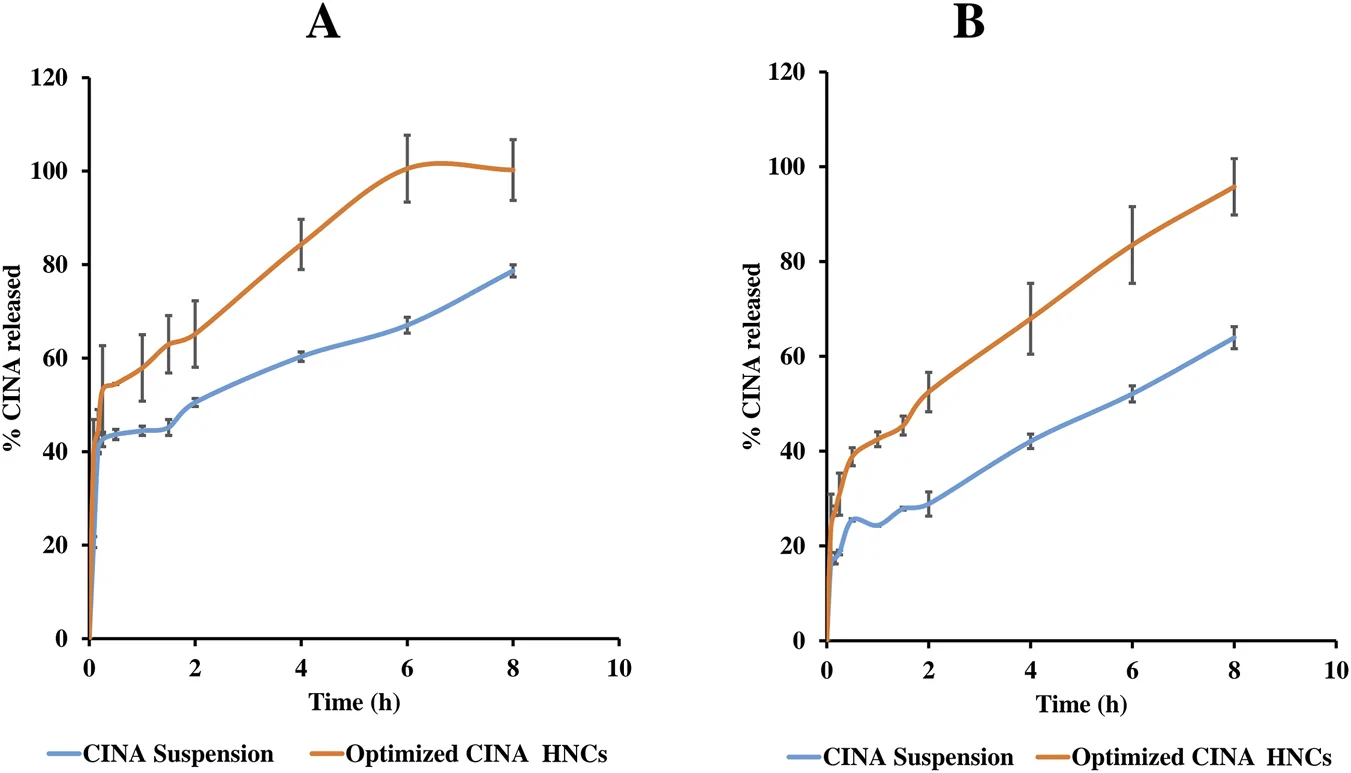
*In vitro* dissolution profiles of the optimized hybrid nanocrystals (HNCs) and the CINA suspension in **(A)** 0.1 N HCl (pH 1.2) and **(B)** phosphate buffer (pH 6.8). Data are presented as mean ± SD (n = 3). The optimized HNCs exhibited a characteristic biphasic release pattern with significantly enhanced drug dissolution compared with the CINA suspension in both dissolution media.

The *in vitro* dissolution performance of the optimized CINA HNCs was comparatively assessed against a plain CINA suspension in two biorelevant media, namely, 0.1 N HCl and phosphate buffer (pH 6.8), over an 8-h period (Figure 2). The optimized formulation demonstrated a markedly superior release profile in both media. Complete drug release (≈100%) was achieved within 6 h in acidic medium and within 8 h in phosphate buffer (pH 6.8). In contrast, the untreated drug suspension exhibited substantially slower and incomplete dissolution, reaching only 78.65% ± 1.30% in 0.1 N HCl and 63.92% ± 2.30% in phosphate buffer after 8 h.

Importantly, the optimized HNCs displayed a distinct biphasic release pattern. The initial phase was characterized by a rapid release within the early time points, which can be attributed to the markedly reduced particle size and the consequent enlargement in effective surface area. Nanonization enhances the dissolution velocity according to diffusion-layer theory, while the increased curvature of nanocrystals may elevate apparent saturation solubility, collectively promoting immediate drug dissolution (Da Silva et al., 2020; Mahmood et al., 2023). Additionally, the presence of hydrophilic stabilizers likely facilitated rapid wetting and dispersion, further accelerating the early release phase. This was followed by a more gradual and sustained release phase, reflecting controlled diffusion of the remaining crystalline core into the dissolution medium. As surface-associated drug dissolved, the release rate became governed by diffusion from the residual nanocrystal matrix, resulting in a moderated dissolution slope. The faster release observed in 0.1 N HCl compared to phosphate buffer (pH 6.8) is consistent with the pH-dependent solubility of CINA, a weakly basic compound that undergoes protonation under acidic conditions, thereby enhancing aqueous solubility. Collectively, these findings confirm that nanocrystal engineering successfully transformed CINA into a gradual and prolonged system with a characteristic biphasic release behavior, a feature that is highly advantageous for improving oral absorption and, consequently, systemic bioavailability (Ragheb et al., 2026; Tung et al., 2022).

Kinetic analysis demonstrated that the optimized HNCs were best described using the Higuchi model in both 0.1 N HCl (R^2^ = 0.9729) and phosphate buffer (pH 6.8) (R^2^ = 0.9907), indicating that drug release was predominantly diffusion-controlled. Moreover, the optimized HNCs exhibited markedly higher dissolution efficiency (DE%) than the conventional CINA suspension in both dissolution media. In acidic medium (0.1 N HCl, pH 1.2), the DE% increased from 58.74% for the CINA suspension to 81.01% for the optimized HNCs. Similarly, in phosphate buffer (pH 6.8), the DE% increased from 41.24% to 66.51%, confirming the superior dissolution performance of the hybrid nanocrystal formulation under both gastric and intestinal conditions.

#### 3.3.2 Saturated solubility in simulated gastrointestinal media

The saturated solubility of the optimized nanocrystals was evaluated in simulated gastric and intestinal media to investigate their behavior under physiological conditions. The optimized formulation exhibited a saturated solubility of 2.24 ± 0.08 mg/mL in simulated gastric fluid (0.1 N HCl, pH 1.2), whereas a lower value of 0.92 ± 0.04 mg/mL was observed in simulated intestinal fluid (phosphate buffer, pH 6.8). The observed pH-dependent solubility profile is consistent with the physicochemical properties of cinacalcet, a weakly basic drug with a pKa of approximately 9.3. Under acidic gastric conditions, extensive protonation of the amine group increases drug ionization, resulting in markedly enhanced aqueous solubility. Conversely, at intestinal pH, the degree of ionization decreases, leading to reduced equilibrium solubility. Despite this expected reduction, the optimized hybrid nanocrystals maintained appreciable drug solubility in intestinal medium, which can be attributed to the combined effects of nanoscale particle size, improved wettability, and stabilization by the polymer–surfactant matrix. These findings demonstrate that the hybrid nanocrystal formulation preserves favorable solubility across physiologically relevant gastrointestinal environments and supports its potential to improve the oral performance of poorly water-soluble cinacalcet.

#### 3.3.3 Transmission electronic microscopy

**FIGURE 3:**
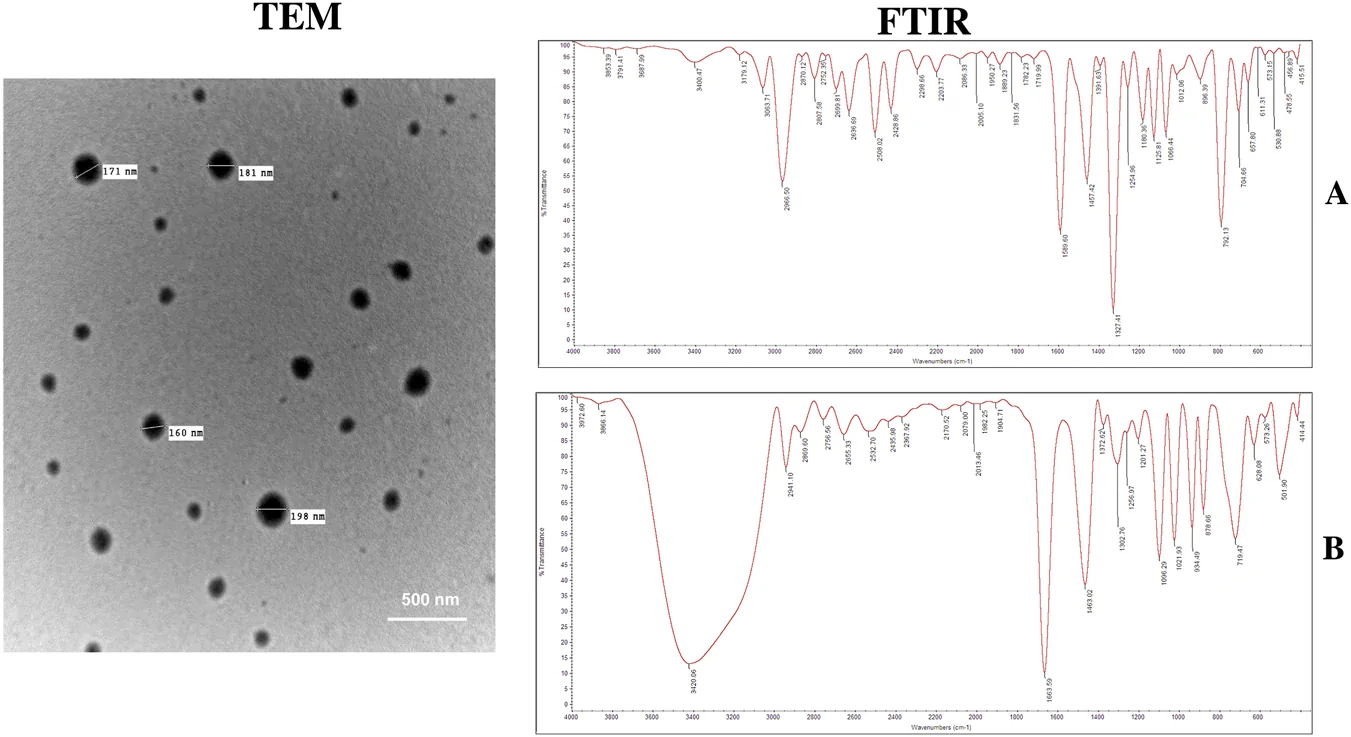
FTIR spectra of **(A)** pure CINA and **(B)** optimum formula. In addition, TEM of the optimum formula, revealing uniformly spherical vesicles with successful drug encapsulation within the nanocrystal matrix.

The TEM micrographs confirmed the formation of discrete, nearly spherical nanocrystals with a narrow size distribution, as shown in Figure 3. The particles appeared uniformly dispersed with no visible aggregation, indicating that the optimized formulation maintained good colloidal stability after preparation. The particle size observed by TEM was consistent with the values obtained by dynamic light scattering, supporting the reliability of the particle size measurements. These findings demonstrate that the selected formulation and processing conditions produced a homogeneous nanocrystal population with the desired morphological characteristics (Sakr et al., 2023; Sayed et al., 2021b).

#### 3.3.4 Zeta potential

Zeta potential represents the total surface charge of particles dispersed in a specific medium and is considered an important physical property influencing colloidal behavior (El Taweel et al., 2023; Ahmed et al., 2026f). It is also a key parameter for predicting the stability of nano-particulate systems (Ozturk et al., 2024). The optimized CINA hybrid nanocrystals exhibited a zeta potential of −24.25 ± 0.64 mV, indicating the presence of a moderate negative surface charge that contributes to dispersion stability. Although this value provides a degree of electrostatic repulsion between particles, the overall stability of the system is mainly attributed to steric stabilization. PVP K90 might act as a physical barrier that prevents direct particle–particle contact and reduces the risk of aggregation. Therefore, the stability of the optimized formulation results from a combination of moderate electrostatic repulsion and effective steric hindrance, ensuring maintenance of nanoscale size and uniform dispersion (Zhou et al., 2023; Wayman et al., 2024).

#### 3.3.5 FTIR

FTIR spectroscopy was performed to evaluate the chemical integrity of cinacalcet after hybrid nanocrystal preparation and to investigate the nature of the interactions between the drug and the stabilizing excipients. The FTIR spectra of raw CINA and the optimized formula are presented in Figure 3. The spectrum of raw CINA exhibited the characteristic absorption bands corresponding to its chemical structure. The aromatic C–H stretching vibration appeared at 3,063.71 cm^−1^, while the aliphatic C–H stretching band was observed at 2,966.50 cm^−1^. The N–H stretching vibration was detected at 3,400.47 cm^−1^, and the aromatic C=C skeletal stretching vibration was recorded at 1,589.60 cm^−1^. In addition, the characteristic asymmetric stretching vibration of the trifluoromethyl (–CF_3_) group was observed at 1,327.41 cm^−1^, whereas the out-of-plane aromatic C–H bending vibrations appeared at 792.13 and 704.66 cm^−1^. These bands agree well with the reported structural features of CINA (Rao et al., 2014).

Following the preparation of the hybrid nanocrystals, the major characteristic absorption bands of CINA remained clearly detectable, confirming that the molecular structure of the drug was preserved throughout the formulation process. Importantly, no disappearance of the characteristic functional groups or appearance of additional absorption bands was observed, indicating that no chemical degradation or covalent modification occurred during nanocrystal fabrication. Nevertheless, several characteristic peaks exhibited noticeable shifts in their positions, suggesting the existence of physical interactions between CINA and the stabilizing matrix. The N–H stretching vibration shifted from 3,400.47 cm^−1^ in the raw drug to 3,420.06 cm^−1^ in the hybrid nanocrystals, accompanied by a marked broadening of the absorption band. This behavior is indicative of intermolecular hydrogen-bond formation between the amine group of CINA and the hydrophilic functional groups of PVP K90 and Pluronic F127. Such hydrogen bonding is expected to enhance the adsorption of the polymers onto the nanocrystal surface, thereby improving particle stabilization. In addition, the aliphatic C–H stretching band shifted from 2,966.50 to 2,941.10 cm^−1^, while the aromatic C=C stretching vibration shifted from 1,589.60 cm^−1^ to 1,463.02 cm^−1^. These red shifts suggest changes in the local molecular environment of the drug, resulting from hydrophobic interactions between the lipophilic regions of cinacalcet and the surfactant molecules, particularly Tween 80 and Pluronic F127. Such interactions contribute to the physical confinement of the drug within the hybrid nanocrystal structure and play an important role in maintaining colloidal stability.

The FTIR spectrum of the hybrid nanocrystals also displayed characteristic absorption bands corresponding to the formulation excipients. A distinct carbonyl (C=O) stretching band of PVP K90 appeared at 1,663.59 cm^−1^, while the ether (C–O–C) stretching vibration of Pluronic F127 and Tween 80 was observed at 1,096.29 cm^−1^. Furthermore, the broad amine salt absorption region observed in raw cinacalcet between 2,870 and 2,428 cm^−1^ was replaced by a broader hydrogen-bonded N–H stretching band centered at 3,420.06 cm^−1^, providing additional evidence for the adsorption of the polymer-surfactant layer onto the nanocrystal surface. Overall, the FTIR findings demonstrate that the hybrid nanocrystals were stabilized through non-covalent intermolecular interactions, predominantly hydrogen bonding together with hydrophobic and steric interactions, rather than through chemical bond formation. The preservation of all characteristic CINA functional groups confirms the excellent compatibility between the drug and the selected stabilizers and indicates that the formulation process maintained the chemical integrity of CINA while promoting effective electro-steric stabilization of the hybrid nanocrystals.

#### 3.3.6 X-ray powder diffraction (XRPD)

**FIGURE 4:**
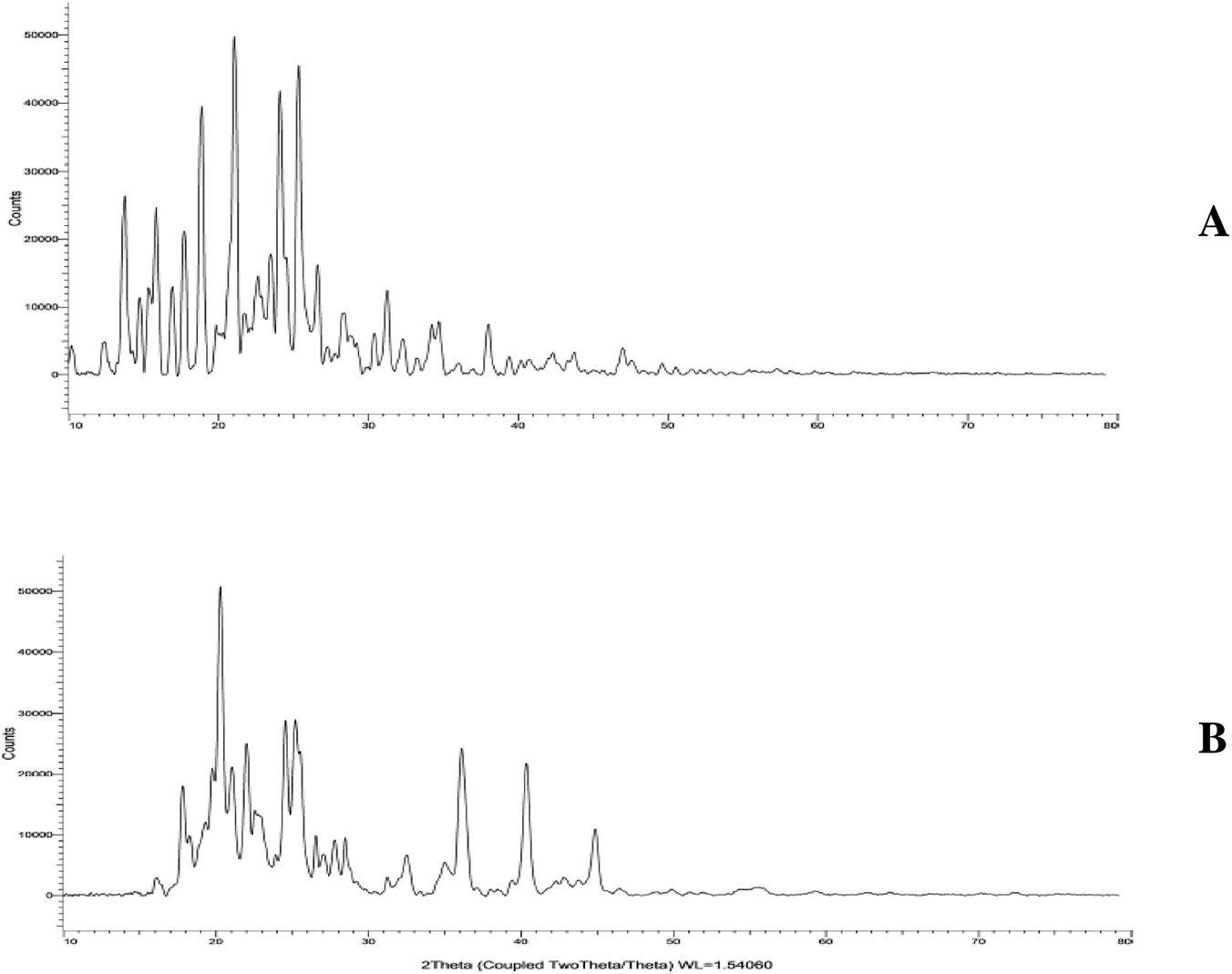
X-ray diffraction analysis of **(A)** pure CINA and **(B)** optimum formula, confirming that CINA retains its crystalline state without conversion to an amorphous form or undergoing polymorphic transformation during high-shear processing.

As shown in Figure 4, the diffractograms of the raw CINA exhibits sharp, intense crystalline reflections, most notably at 2θ values at approximately 13.5°, 15.5°, 17.6°, 18.8°, 20.4°, 24.1°, and 25.3°, confirming its highly crystalline nature. The X-ray powder diffraction (XRPD) pattern of the optimized HNCs preserves these key characteristic diffraction peaks (e.g., at 2θ ≈ 17.6°, 20.4°, 24.7°, and 25.3°), unequivocally confirming that CINA retains its crystalline state without conversion to an amorphous form or undergoing polymorphic transformation during high-shear processing. A slight reduction in peak intensities and slight peak broadening in HNCs are expected consequences of particle size reduction to the nanoscale (Scherrer line broadening effect) and surface coverage by amorphous excipients (PVP K90/Tween 80).

#### 3.3.7 Stability assessment

The optimized hybrid nanosystem exhibited excellent physicochemical stability under both refrigerated and accelerated storage conditions. Comparative analysis between freshly prepared and stored samples revealed no statistically significant differences (p > 0.05) in PS, PDI, ZP, or SS (Table 5), confirming the preservation of the formulation’s critical quality attributes and supporting its suitability for oral delivery. The observed stability can be primarily attributed to the combined electrostatic and steric stabilization mechanisms within the system. The maintained zeta potential indicates the presence of sufficient surface charge to generate electrostatic repulsion between adjacent particles, thereby minimizing attractive forces that could otherwise promote aggregation (Fahmy et al., 2025; Ahmed et al., 2026g). This electrostatic barrier plays an essential role in preserving nanoscale dispersion uniformity over time. In addition, PVP functions as an effective steric stabilizer. The polymer adsorbs onto the particle surface through intermolecular interactions and extends its hydrophilic chains into the surrounding aqueous environment, forming a hydrated protective layer. This steric barrier prevents close particle–particle contact and reduces the probability of coalescence or growth during storage (Zhou et al., 2023).

**TABLE 5:** Post-storage evaluation of the optimized formula.

Parameter	Fresh	Storage for 3 months at 4 °C–8 °C		Storage for 1 month at 40 °C, 75% RH	
		Value	Probability (p)*	Value	Probability (p)*
PS (nm)	193.44 ± 3.44	201.51 ± 4.22	0.171	197.14 ± 1.41	0.295
PDI	0.34 ± 0.02	0.36 ± 0.01	0.333	0.35 ± 0.01	0.591
ZP (V)	−24.25 ± 0.64	−21.65 ± 2.51	0.260	−19.83 ± 3.47	0.202
SS (mg/mL)	2.04 ± 0.03	2.01 ± 0.02	0.360	2.02 ± 0.01	0.465

Abbreviations: PDI, poly-dispersity index; PS, particle size; SS, saturated solubility; ZP, zeta potential.

* One-way ANOVA analysis to compare between the freshly prepared and the stored optimum formula.

### 3.4 *In vivo* pharmacokinetics

Cinacalcet is a weakly basic, highly lipophilic drug (pKa ≈ 9.3; log P ≈ 4.7) whose oral absorption is known to be influenced by food intake. Clinically, administration with food, particularly a high-fat meal, has been reported to increase both the Cmax and overall systemic exposure (AUC), mainly due to enhanced drug solubilization by bile salts and mixed micelles, together with prolonged gastric residence time that favors drug dissolution in the acidic gastric environment before intestinal transit. Therefore, the *in vivo* pharmacokinetic study was conducted under fed conditions to closely simulate the recommended clinical administration of cinacalcet.

**FIGURE 5:**
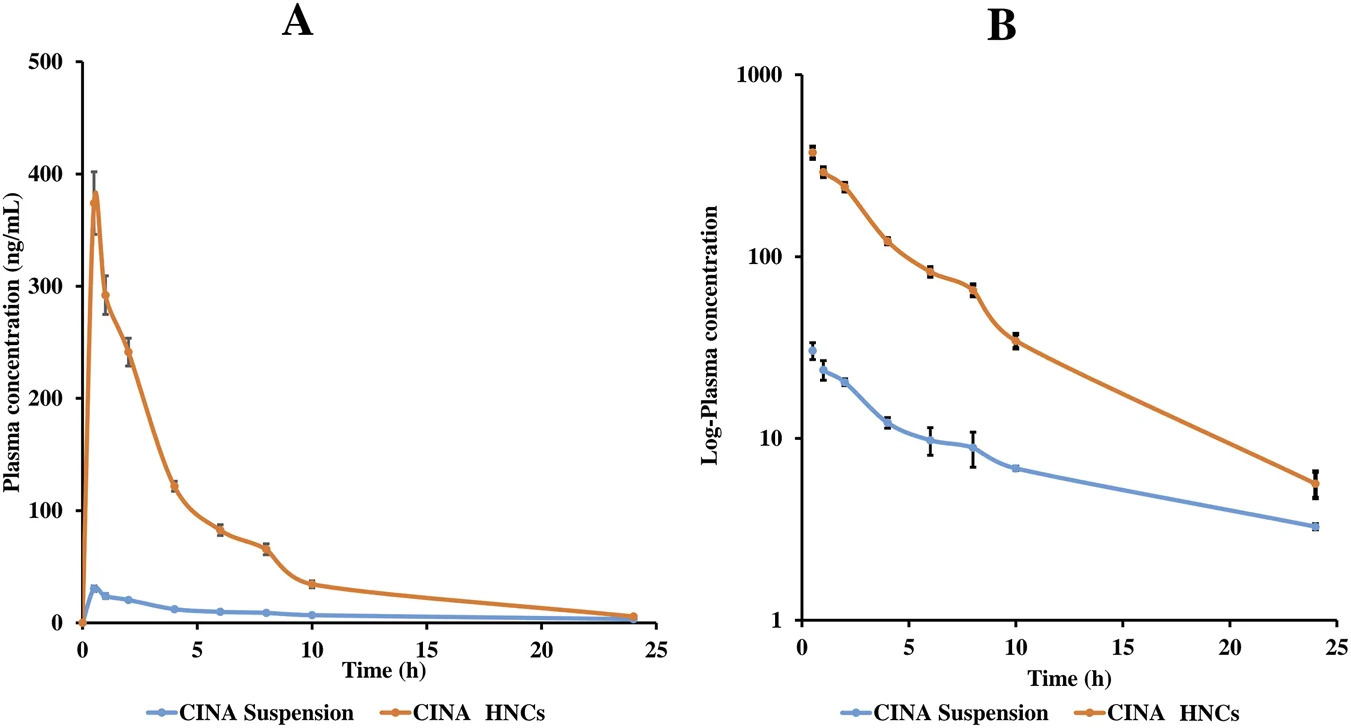
**(A)** Plasma concentration–time profiles **(B)** Log-plasma concentration–time profiles of cinacalcet (CINA) following oral administration of the optimized hybrid nanocrystals (HNCs) and the CINA suspension in rabbits. Data are presented as mean ± SD (n = 4). The optimized HNCs produced markedly higher plasma concentrations throughout the study, resulting in significantly greater systemic exposure than the CINA suspension.

**FIGURE 6:**
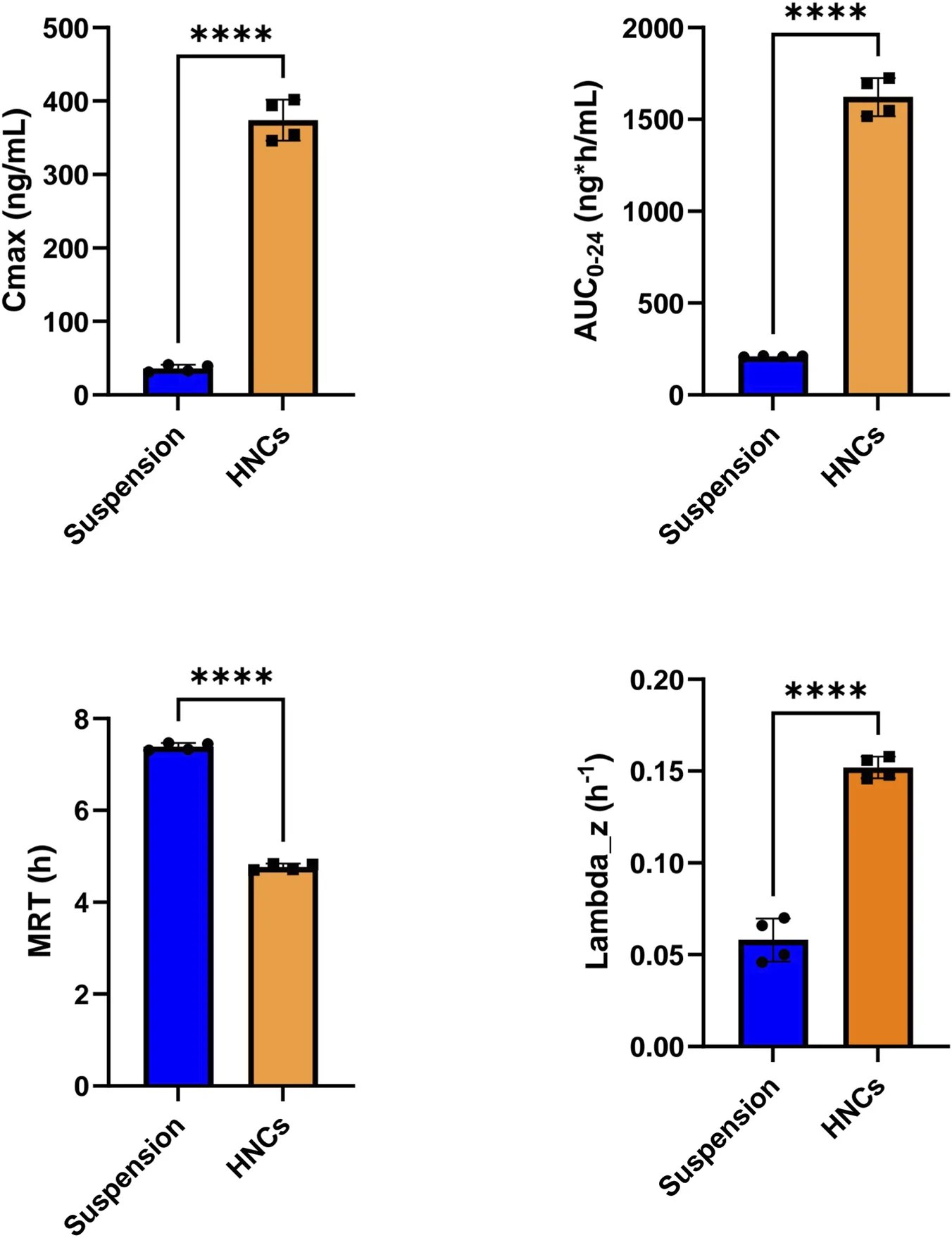
Statistical comparison of the pharmacokinetic parameters of the optimized hybrid nanocrystals (HNCs) and the CINA suspension. Data are presented as mean ± SD (n = 4). Statistical analysis was performed using Student's t-test. Statistical significance was defined as *p < 0.05, **p < 0.01, ***p < 0.001, and ****p < 0.0001.

The plasma concentration–time curves of CINA following oral administration of the optimized HNCs and the conventional drug suspension are illustrated in Figure 5, while the corresponding pharmacokinetic parameters are summarized in Table 6 and Figure 6. The obtained data clearly demonstrate a substantial enhancement in the oral pharmacokinetic performance of CINA after formulation as hybrid nanocrystals compared with the conventional suspension. The optimized CINA HNCs achieved a markedly higher Cmax of 374.08 ± 27.84 ng/mL, representing approximately a 10.36-fold increase relative to the raw suspension (36.09 ± 4.88 ng/mL). This pronounced elevation in systemic exposure was further confirmed by the significant increase in AUC_0–24_, which reached 1,621.71 ± 104.91 ng mL^−1^·h for the HNC formulation compared with 208.92 ± 2.87 ng mL^−1^·h for the conventional suspension. Accordingly, the optimized HNCs exhibited a relative bioavailability (F_rel_) of 776.17%, corresponding to a 7.76-fold increase over the conventional suspension. These findings collectively indicate a remarkable improvement in the overall extent of CINA absorption and oral bioavailability following nanocrystal formulation. Interestingly, the time required to reach Tmax remained identical for both formulations (0.5 h), suggesting that the nanocrystal system primarily enhanced the magnitude of drug absorption rather than altering its onset. In addition, the optimized nanocrystals exhibited a shorter MRT (4.77 ± 0.08 h) than the conventional suspension (7.39 ± 0.08 h), accompanied by a relatively higher terminal elimination rate constant (λz), reflecting a more efficient and rapid drug absorption, followed by subsequent distribution and elimination.

**TABLE 6:** *In vivo* pharmacokinetic parameters of the optimized formula compared to the conventional suspension (n = 4, mean ± SD).

Parameter	Conventional suspension	Optimized HNCs
Cmax (ng/mL)****	36.09 ± 4.88	374.08 ± 27.84
Tmax (h)	0.5 ± 0	0.5 ± 0
AUC (h.ng/mL)****	208.92 ± 2.87	1,621.71 ± 104.91
MRT (h)****	7.39 ± 0.08	4.77 ± 0.07
t1/2 (h)****	5.12 ± 0.05	3.31 ± 0.06
Lambda-Z (h^−1^)****	0.058 ± 0.012	0.152 ± 0.006

*p < 0.05, **p < 0.01, ***p < 0.001, and ****p < 0.0001.

Notably, despite the presence of physiological postprandial solubilization mechanisms, the optimized hybrid nanocrystals still produced significantly higher Cmax and AUC values than the pure drug suspension (p < 0.05). These findings indicate that the improved oral bioavailability cannot be attributed solely to the food effect, but rather to the superior dissolution characteristics imparted by the hybrid nanocrystal formulation. The superior pharmacokinetic performance of the HNCs can be attributed to the nanoscale particle size, which substantially increases the specific surface area and promotes rapid dissolution of CINA. This improvement in dissolution behavior enhances the apparent solubility and establishes a stronger concentration gradient across the intestinal membrane, thereby facilitating more efficient drug absorption (Farag et al., 2022). Additionally, the stabilizing agents surrounding the nanocrystals improve wettability and prevent particle aggregation, ensuring consistent dispersion within the gastrointestinal environment. Collectively, these factors contribute to the effective translation of enhanced *in vitro* dissolution into significantly improved *in vivo* oral bioavailability.

It is important to note that species-specific differences in gastrointestinal physiology, including gastric pH, gastrointestinal transit time, intestinal surface area, bile salt composition, and drug-metabolizing enzyme expression, may influence the oral absorption and pharmacokinetic behavior of cinacalcet. Consequently, the magnitude of the bioavailability enhancement observed in rabbits may not directly translate to humans. In addition, although allometric scaling is commonly applied to estimate certain pharmacokinetic parameters across species, it does not adequately account for formulation-dependent absorption processes and therefore was beyond the scope of the present study.

## 4 Limitations of the study

PS, PDI, and ZP measurements were performed at 25 °C under standard laboratory conditions. Future studies should investigate the effect of physiological temperature (37 °C) on these physicochemical properties. Differential Scanning Calorimetry (DSC) analysis could not be performed because the instrument was unavailable due to a technical malfunction during the study period. Consequently, thermal behavior of CINA before and after formulation could not be comprehensively evaluated and warrant further investigation. Additionally, although the observed differences in the pharmacokinetic parameters, including AUC and Cmax, were statistically significant (p < 0.05), the relatively small sample size (n = 4) makes the pharmacokinetic study exploratory and may limit the detection of subtle differences and the assessment of inter-individual variability. Therefore, larger confirmatory pharmacokinetic studies using appropriately powered animal cohorts are recommended to validate these findings before clinical translation. The effect of formulation viscosity on particle size reduction was not directly evaluated by rheological measurements. Moreover, freeze–thaw stability was not evaluated in the present study and should be investigated in future work to further assess the robustness of the optimized formulation under repeated temperature fluctuations.

## 5 Conclusion

In this study, we describe the development of HNCs of cinacalcet to improve the oral delivery of this poorly soluble and poorly permeable BCS Class IV drug. The formulation employed a dual-stabilization approach combining steric and dispersing stabilizers to control nanocrystal formation and maintain colloidal stability. The optimized formulation exhibited a PS of 193.44 nm and a PDI of 0.34. TEM showed discrete, uniformly distributed nanocrystals, while zeta potential measurements and stability studies indicated the maintenance of the physicochemical characteristics during the evaluated storage period. Dissolution studies demonstrated faster and more extensive drug release than the conventional suspension in both simulated gastric (pH 1.2) and intestinal (pH 6.8) media. *In vivo* pharmacokinetic evaluation showed that the optimized HNCs produced a 10.36-fold increase in Cmax and a 7.76-fold increase in AUC compared with the conventional suspension, indicating increased systemic exposure following oral administration. Overall, these findings suggest that hybrid-stabilized nanocrystals represent a suitable formulation approach for improving the oral delivery of cinacalcet.

## Data Availability

The original contributions presented in the study are included in the article/Supplementary Material, further inquiries can be directed to the corresponding authors.
